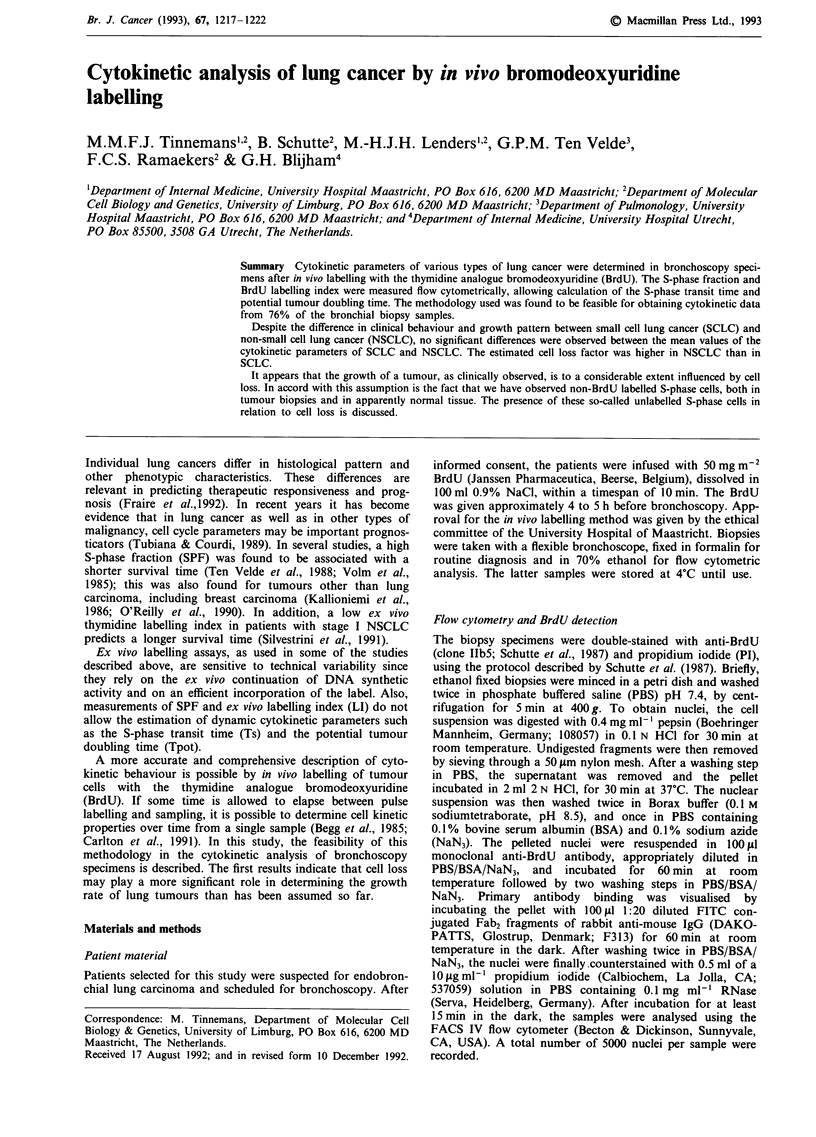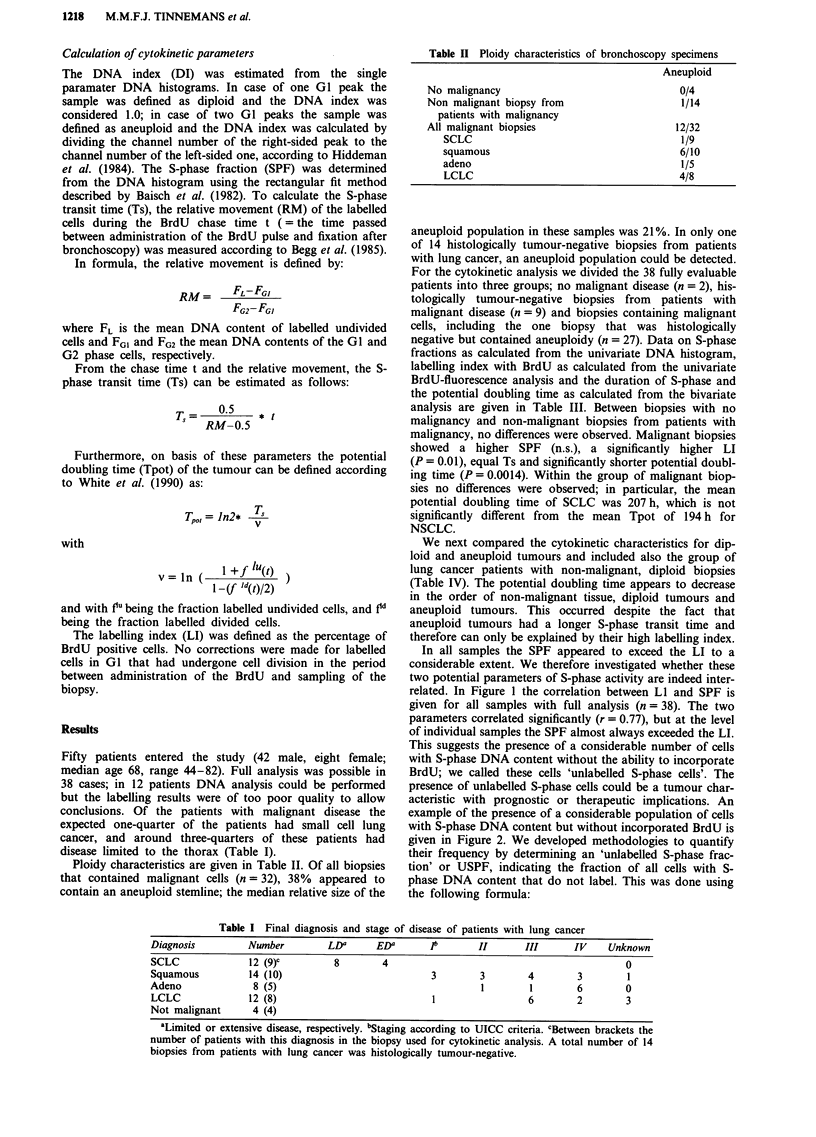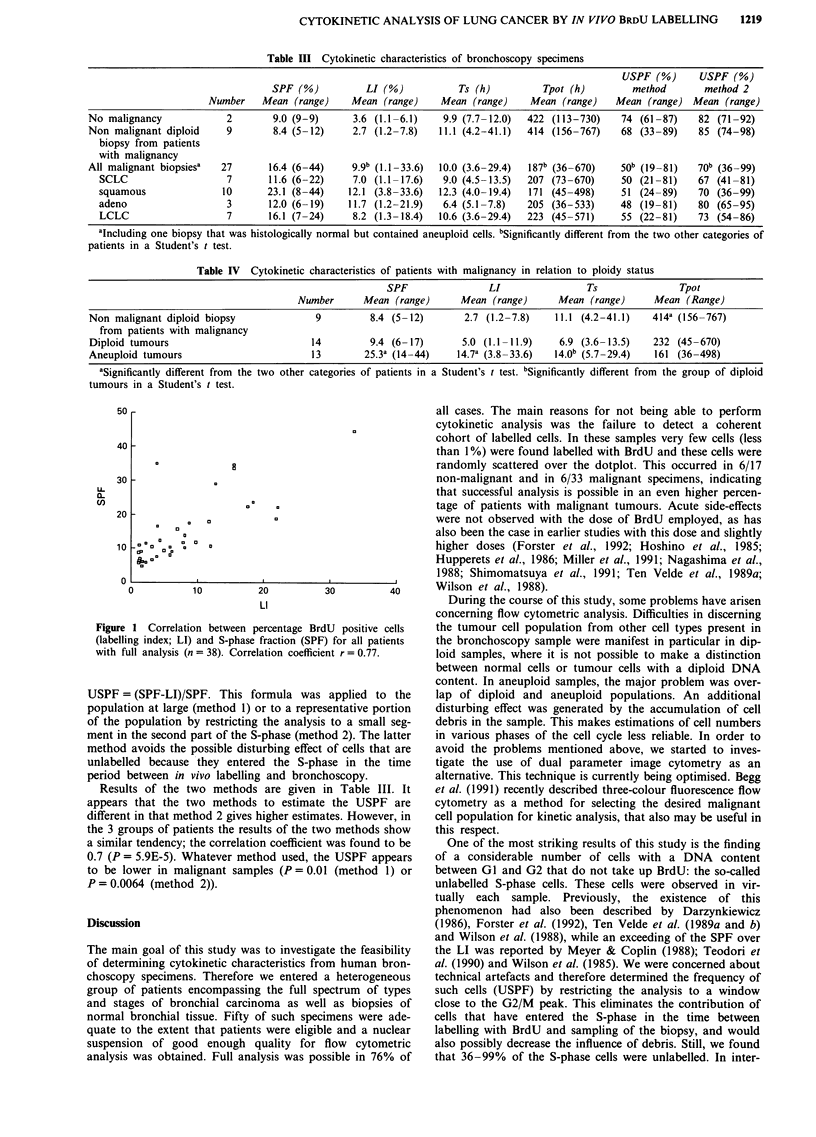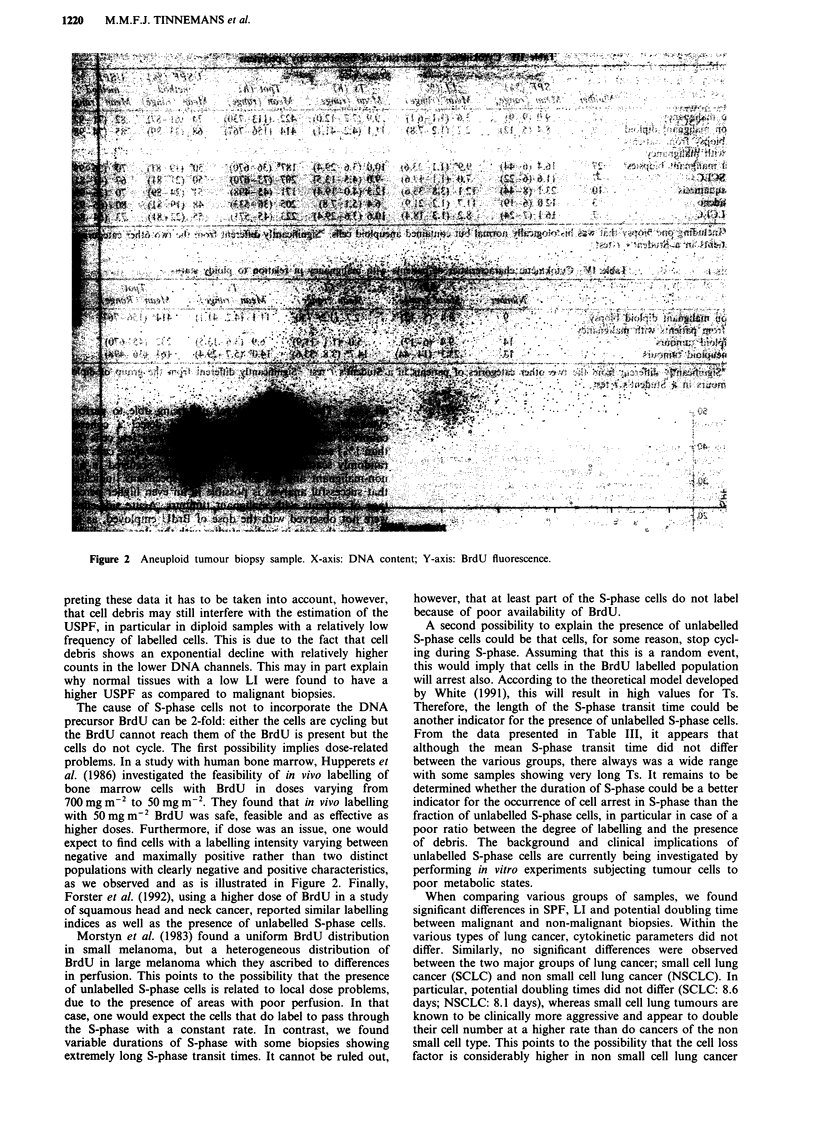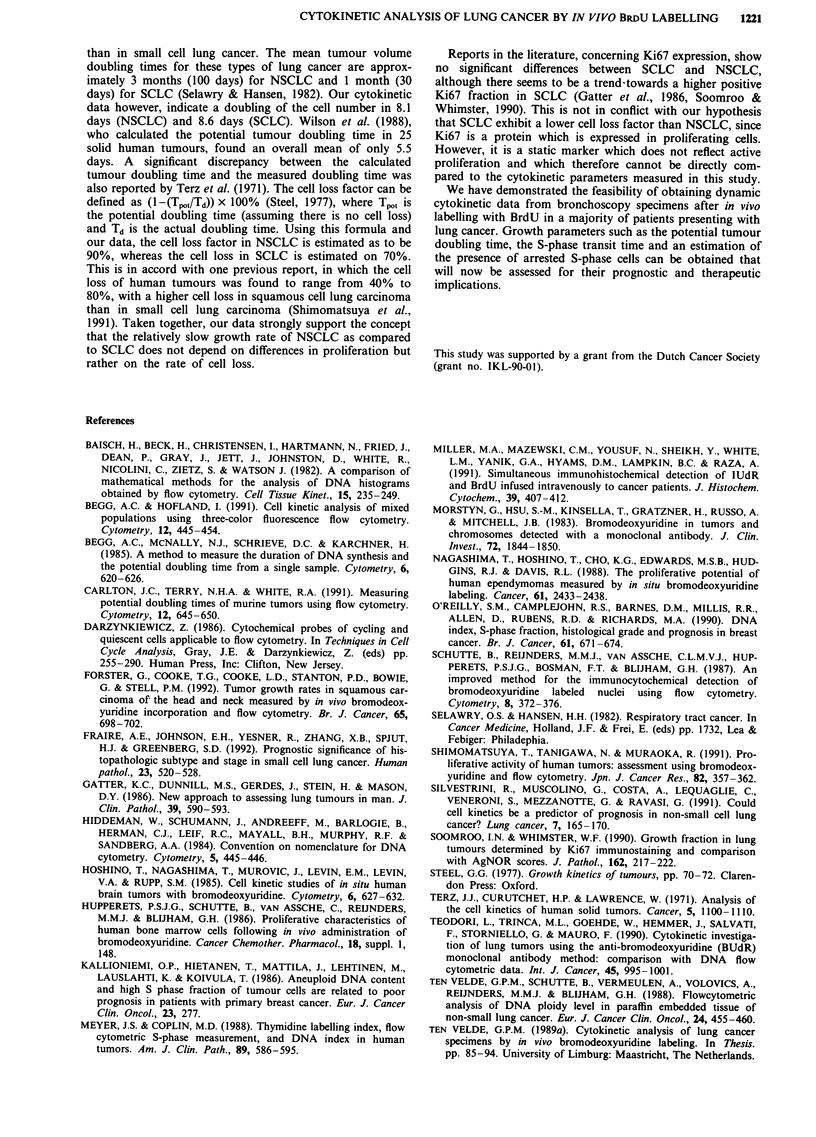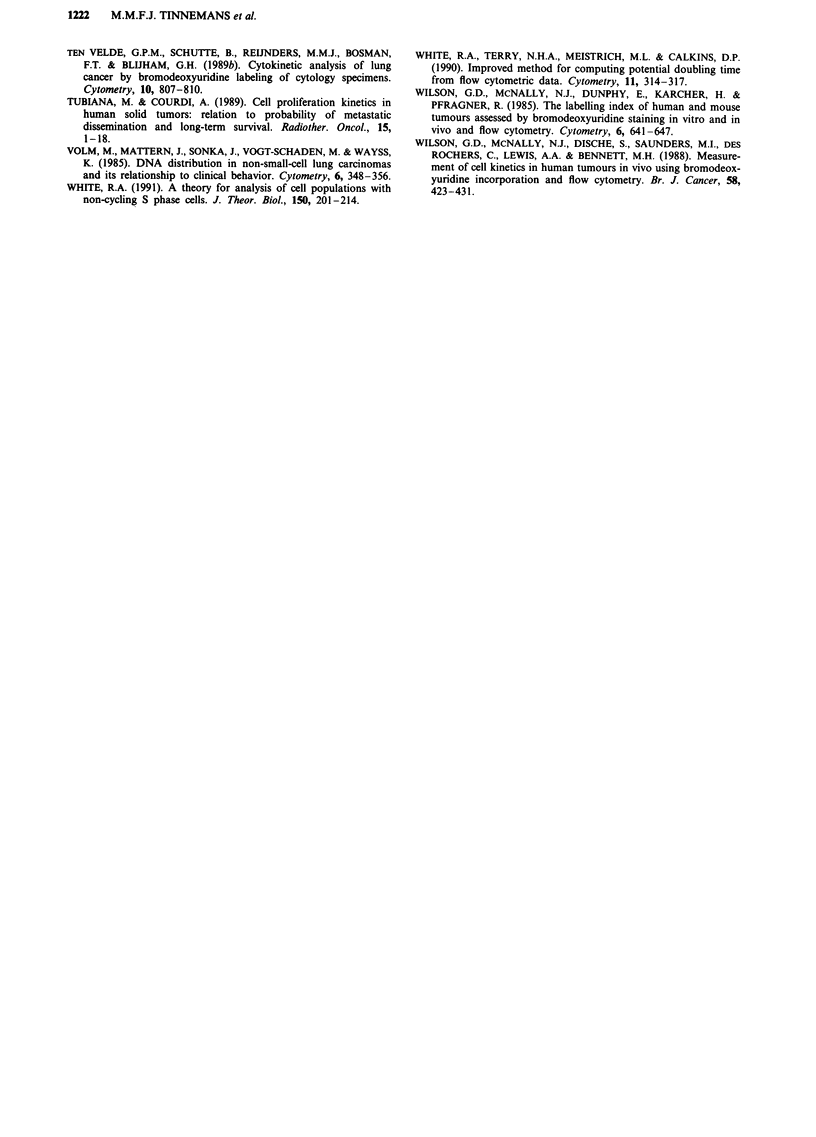# Cytokinetic analysis of lung cancer by in vivo bromodeoxyuridine labelling.

**DOI:** 10.1038/bjc.1993.228

**Published:** 1993-06

**Authors:** M. M. Tinnemans, B. Schutte, M. H. Lenders, G. P. Ten Velde, F. C. Ramaekers, G. H. Blijham

**Affiliations:** Department of Internal Medicine, University Hospital Maastricht, The Netherlands.

## Abstract

Cytokinetic parameters of various types of lung cancer were determined in bronchoscopy specimens after in vivo labelling with the thymidine analogue bromodeoxyuridine (BrdU). The S-phase fraction and BrdU labelling index were measured flow cytometrically, allowing calculation of the S-phase transit time and potential tumour doubling time. The methodology used was found to be feasible for obtaining cytokinetic data from 76% of the bronchial biopsy samples. Despite the difference in clinical behaviour and growth pattern between small cell lung cancer (SCLC) and non-small cell lung cancer (NSCLC), no significant differences were observed between the mean values of the cytokinetic parameters of SCLC and NSCLC. The estimated cell loss factor was higher in NSCLC than in SCLC. It appears that the growth of a tumour, as clinically observed, is to a considerable extent influenced by cell loss. In accord with this assumption is the fact that we have observed non-BrdU labelled S-phase cells, both in tumour biopsies and in apparently normal tissue. The presence of these so-called unlabelled S-phase cells in relation to cell loss is discussed.


Br. J. Cancer (1993), 67, 1217-1222 © Macmillan Press Ltd., 1993

### Cytokinetic analysis of lung cancer by *in vivo* bromodeoxyuridine labelling

M.M.F.J. Tinnemans[1,2], B. Schutte[2], M.-H.J.H. Lenders[1,2], G.P.M. Ten Velde[3], F.C.S. Ramaekers[2] & G.H. Blijham[4]

[1]*Department of Internal Medicine, University Hospital Maastricht, PO Box 616, 6200 MD Maastricht;* [2]*Department of Molecular Cell Biology and Genetics, University of Limburg, PO Box 616, 6200 MD Maastricht;* [3]*Department of Pulmonology, University Hospital Maastricht, PO Box 616, 6200 MD Maastricht; and* [4]*Department of Internal Medicine, University Hospital Utrecht, PO Box 85500, 3508 GA Utrecht, The Netherlands.*

**Summary** Cytokinetic parameters of various types of lung cancer were determined in bronchoscopy specimens after *in vivo* labelling with the thymidine analogue bromodeoxyuridine (BrdU). The S-phase fraction and BrdU labelling index were measured flow cytometrically, allowing calculation of the S-phase transit time and potential tumour doubling time. The methodology used was found to be feasible for obtaining cytokinetic data from 76% of the bronchial biopsy samples.

Despite the difference in clinical behaviour and growth pattern between small cell lung cancer (SCLC) and non-small cell lung cancer (NSCLC), no significant differences were observed between the mean values of the cytokinetic parameters of SCLC and NSCLC. The estimated cell loss factor was higher in NSCLC than in SCLC.

It appears that the growth of a tumour, as clinically observed, is to a considerable extent influenced by cell loss. In accord with this assumption is the fact that we have observed non-BrdU labelled S-phase cells, both in tumour biopsies and in apparently normal tissue. The presence of these so-called unlabelled S-phase cells in relation to cell loss is discussed.

Individual lung cancers differ in histological pattern and other phenotypic characteristics. These differences are relevant in predicting therapeutic responsiveness and prognosis (Fraire *et al.*,1992). In recent years it has become evidence that in lung cancer as well as in other types of malignancy, cell cycle parameters may be important prognosticators (Tubiana & Courdi, 1989). In several studies, a high S-phase fraction (SPF) was found to be associated with a shorter survival time (Ten Velde *et al.*, 1988; Volm *et al.*, 1985); this was also found for tumours other than lung carcinoma, including breast carcinoma (Kallioniemi *et al.*, 1986; O'Reilly *et al.*, 1990). In addition, a low *ex vivo* thymidine labelling index in patients with stage I NSCLC predicts a longer survival time (Silvestrini *et al.*, 1991).

*Ex vivo* labelling assays, as used in some of the studies described above, are sensitive to technical variability since they rely on the *ex vivo* continuation of DNA synthetic activity and on an efficient incorporation of the label. Also, measurements of SPF and *ex vivo* labelling index (LI) do not allow the estimation of dynamic cytokinetic parameters such as the S-phase transit time (Ts) and the potential tumour doubling time (Tpot).

A more accurate and comprehensive description of cytokinetic behaviour is possible by *in vivo* labelling of tumour cells with the thymidine analogue bromodeoxyuridine (BrdU). If some time is allowed to elapse between pulse labelling and sampling, it is possible to determine cell kinetic properties over time from a single sample (Begg *et al.*, 1985; Carlton *et al.*, 1991). In this study, the feasibility of this methodology in the cytokinetic analysis of bronchoscopy specimens is described. The first results indicate that cell loss may play a more significant role in determining the growth rate of lung tumours than has been assumed so far.

### Materials and methods

### *Patient material*

Patients selected for this study were suspected for endobronchial lung carcinoma and scheduled for bronchoscopy. After informed consent, the patients were infused with 50 mg $m^{-2}$ BrdU (Janssen Pharmaceutica, Beerse, Belgium), dissolved in 100 ml 0.9% NaCl, within a timespan of 10 min. The BrdU was given approximately 4 to 5 h before bronchoscopy. Approval for the *in vivo* labelling method was given by the ethical committee of the University Hospital of Maastricht. Biopsies were taken with a flexible bronchoscope, fixed in formalin for routine diagnosis and in 70% ethanol for flow cytometric analysis. The latter samples were stored at 4°C until use.

### *Flow cytometry and BrdU detection*

The biopsy specimens were double-stained with anti-BrdU (clone IIb5; Schutte *et al.*, 1987) and propidium iodide (PI), using the protocol described by Schutte *et al.* (1987). Briefly, ethanol fixed biopsies were minced in a petri dish and washed twice in phosphate buffered saline (PBS) pH 7.4, by centrifugation for 5 min at 400 *g*. To obtain nuclei, the cell suspension was digested with 0.4 mg $ml^{-1}$ pepsin (Boehringer Mannheim, Germany; 108057) in 0.1 N HCl for 30 min at room temperature. Undigested fragments were then removed by sieving through a 50 $\mu m$ nylon mesh. After a washing step in PBS, the supernatant was removed and the pellet incubated in 2 ml 2 N HCl, for 30 min at 37°C. The nuclear suspension was then washed twice in Borax buffer (0.1 M sodiumtetraborate, pH 8.5), and once in PBS containing 0.1% bovine serum albumin (BSA) and 0.1% sodium azide ($NaN_3$). The pelleted nuclei were resuspended in 100 $\mu l$ monoclonal anti-BrdU antibody, appropriately diluted in PBS/BSA/$NaN_3$, and incubated for 60 min at room temperature followed by two washing steps in PBS/BSA/$NaN_3$. Primary antibody binding was visualised by incubating the pellet with 100 $\mu l$ 1:20 diluted FITC conjugated $Fab_2$ fragments of rabbit anti-mouse IgG (DAKOPATTS, Glostrup, Denmark; F313) for 60 min at room temperature in the dark. After washing twice in PBS/BSA/$NaN_3$, the nuclei were finally counterstained with 0.5 ml of a 10 $\mu g$ $ml^{-1}$ propidium iodide (Calbiochem, La Jolla, CA; 537059) solution in PBS containing 0.1 mg $ml^{-1}$ RNase (Serva, Heidelberg, Germany). After incubation for at least 15 min in the dark, the samples were analysed using the FACS IV flow cytometer (Becton & Dickinson, Sunnyvale, CA, USA). A total number of 5000 nuclei per sample were recorded.

Correspondence: M. Tinnemans, Department of Molecular Cell Biology & Genetics, University of Limburg, PO Box 616, 6200 MD Maastricht, The Netherlands.
Received 17 August 1992; and in revised form 10 December 1992.

*Calculation of cytokinetic parameters*

The DNA index (DI) was estimated from the single paramater DNA histograms. In case of one G1 peak the sample was defined as diploid and the DNA index was considered 1.0; in case of two G1 peaks the sample was defined as aneuploid and the DNA index was calculated by dividing the channel number of the right-sided peak to the channel number of the left-sided one, according to Hiddeman *et al.* (1984). The S-phase fraction (SPF) was determined from the DNA histogram using the rectangular fit method described by Baisch *et al.* (1982). To calculate the S-phase transit time (Ts), the relative movement (RM) of the labelled cells during the BrdU chase time t ( = the time passed between administration of the BrdU pulse and fixation after bronchoscopy) was measured according to Begg *et al.* (1985).

In formula, the relative movement is defined by:

$$RM = \frac{F_L - F_{G1}}{F_{G2} - F_{G1}}$$

where $F_L$ is the mean DNA content of labelled undivided cells and $F_{G1}$ and $F_{G2}$ the mean DNA contents of the G1 and G2 phase cells, respectively.

From the chase time t and the relative movement, the S-phase transit time (Ts) can be estimated as follows:

$$T_s = \frac{0.5}{RM - 0.5} * t$$

Furthermore, on basis of these parameters the potential doubling time (Tpot) of the tumour can be defined according to White *et al.* (1990) as:

$$T_{pot} = ln2 * \frac{T_s}{v}$$

with

$$v = \ln\left(\frac{1 + f^{lu}(t)}{1 - (f^{ld}(t)/2)}\right)$$

and with $f^{lu}$ being the fraction labelled undivided cells, and $f^{ld}$ being the fraction labelled divided cells.

The labelling index (LI) was defined as the percentage of BrdU positive cells. No corrections were made for labelled cells in G1 that had undergone cell division in the period between administration of the BrdU and sampling of the biopsy.

### Results

Fifty patients entered the study (42 male, eight female; median age 68, range 44–82). Full analysis was possible in 38 cases; in 12 patients DNA analysis could be performed but the labelling results were of too poor quality to allow conclusions. Of the patients with malignant disease the expected one-quarter of the patients had small cell lung cancer, and around three-quarters of these patients had disease limited to the thorax (Table I).

Ploidy characteristics are given in Table II. Of all biopsies that contained malignant cells ($n = 32$), 38% appeared to contain an aneuploid stemline; the median relative size of the aneuploid population in these samples was 21%. In only one of 14 histologically tumour-negative biopsies from patients with lung cancer, an aneuploid population could be detected. For the cytokinetic analysis we divided the 38 fully evaluable patients into three groups; no malignant disease ($n = 2$), histologically tumour-negative biopsies from patients with malignant disease ($n = 9$) and biopsies containing malignant cells, including the one biopsy that was histologically negative but contained aneuploidy ($n = 27$). Data on S-phase fractions as calculated from the univariate DNA histogram, labelling index with BrdU as calculated from the univariate BrdU-fluorescence analysis and the duration of S-phase and the potential doubling time as calculated from the bivariate analysis are given in Table III. Between biopsies with no malignancy and non-malignant biopsies from patients with malignancy, no differences were observed. Malignant biopsies showed a higher SPF (n.s.), a significantly higher LI ($P = 0.01$), equal Ts and significantly shorter potential doubling time ($P = 0.0014$). Within the group of malignant biopsies no differences were observed; in particular, the mean potential doubling time of SCLC was 207 h, which is not significantly different from the mean Tpot of 194 h for NSCLC.

**Table II** Ploidy characteristics of bronchoscopy specimens

| | Aneuploid |
|---|---|
| No malignancy | 0/4 |
| Non malignant biopsy from patients with malignancy | 1/14 |
| All malignant biopsies | 12/32 |
| SCLC | 1/9 |
| squamous | 6/10 |
| adeno | 1/5 |
| LCLC | 4/8 |

We next compared the cytokinetic characteristics for diploid and aneuploid tumours and included also the group of lung cancer patients with non-malignant, diploid biopsies (Table IV). The potential doubling time appears to decrease in the order of non-malignant tissue, diploid tumours and aneuploid tumours. This occurred despite the fact that aneuploid tumours had a longer S-phase transit time and therefore can only be explained by their high labelling index.

In all samples the SPF appeared to exceed the LI to a considerable extent. We therefore investigated whether these two potential parameters of S-phase activity are indeed interrelated. In Figure 1 the correlation between L1 and SPF is given for all samples with full analysis ($n = 38$). The two parameters correlated significantly ($r = 0.77$), but at the level of individual samples the SPF almost always exceeded the LI. This suggests the presence of a considerable number of cells with S-phase DNA content without the ability to incorporate BrdU; we called these cells 'unlabelled S-phase cells'. The presence of unlabelled S-phase cells could be a tumour characteristic with prognostic or therapeutic implications. An example of the presence of a considerable population of cells with S-phase DNA content but without incorporated BrdU is given in Figure 2. We developed methodologies to quantify their frequency by determining an 'unlabelled S-phase fraction' or USPF, indicating the fraction of all cells with S-phase DNA content that do not label. This was done using the following formula:

**Table I** Final diagnosis and stage of disease of patients with lung cancer

| *Diagnosis* | *Number* | *LD*[a] | *ED*[a] | *I*[b] | *II* | *III* | *IV* | *Unknown* |
|---|---|---|---|---|---|---|---|---|
| SCLC | 12 (9)[c] | 8 | 4 | | | | | 0 |
| Squamous | 14 (10) | | | 3 | 3 | 4 | 3 | 1 |
| Adeno | 8 (5) | | | | 1 | 1 | 6 | 0 |
| LCLC | 12 (8) | | | 1 | | 6 | 2 | 3 |
| Not malignant | 4 (4) | | | | | | | |

[a]Limited or extensive disease, respectively. [b]Staging according to UICC criteria. [c]Between brackets the number of patients with this diagnosis in the biopsy used for cytokinetic analysis. A total number of 14 biopsies from patients with lung cancer was histologically tumour-negative.

**Table III** Cytokinetic characteristics of bronchoscopy specimens

| | *Number* | *SPF (%) Mean (range)* | *LI (%) Mean (range)* | *Ts (h) Mean (range)* | *Tpot (h) Mean (range)* | *USPF (%) method Mean (range)* | *USPF (%) method 2 Mean (range)* |
|---|---|---|---|---|---|---|---|
| No malignancy | 2 | 9.0 (9–9) | 3.6 (1.1–6.1) | 9.9 (7.7–12.0) | 422 (113–730) | 74 (61–87) | 82 (71–92) |
| Non malignant diploid biopsy from patients with malignancy | 9 | 8.4 (5–12) | 2.7 (1.2–7.8) | 11.1 (4.2–41.1) | 414 (156–767) | 68 (33–89) | 85 (74–98) |
| All malignant biopsies[a] | 27 | 16.4 (6–44) | 9.9[b] (1.1–33.6) | 10.0 (3.6–29.4) | 187[b] (36–670) | 50[b] (19–81) | 70[b] (36–99) |
| SCLC | 7 | 11.6 (6–22) | 7.0 (1.1–17.6) | 9.0 (4.5–13.5) | 207 (73–670) | 50 (21–81) | 67 (41–81) |
| squamous | 10 | 23.1 (8–44) | 12.1 (3.8–33.6) | 12.3 (4.0–19.4) | 171 (45–498) | 51 (24–89) | 70 (36–99) |
| adeno | 3 | 12.0 (6–19) | 11.7 (1.2–21.9) | 6.4 (5.1–7.8) | 205 (36–533) | 48 (19–81) | 80 (65–95) |
| LCLC | 7 | 16.1 (7–24) | 8.2 (1.3–18.4) | 10.6 (3.6–29.4) | 223 (45–571) | 55 (22–81) | 73 (54–86) |

[a]Including one biopsy that was histologically normal but contained aneuploid cells. [b]Significantly different from the two other categories of patients in a Student's *t* test.

**Table IV** Cytokinetic characteristics of patients with malignancy in relation to ploidy status

| | *Number* | *SPF Mean (range)* | *LI Mean (range)* | *Ts Mean (range)* | *Tpot Mean (Range)* |
|---|---|---|---|---|---|
| Non malignant diploid biopsy from patients with malignancy | 9 | 8.4 (5–12) | 2.7 (1.2–7.8) | 11.1 (4.2–41.1) | 414[a] (156–767) |
| Diploid tumours | 14 | 9.4 (6–17) | 5.0 (1.1–11.9) | 6.9 (3.6–13.5) | 232 (45–670) |
| Aneuploid tumours | 13 | 25.3[a] (14–44) | 14.7[a] (3.8–33.6) | 14.0[b] (5.7–29.4) | 161 (36–498) |

[a]Significantly different from the two other categories of patients in a Student's *t* test. [b]Significantly different from the group of diploid tumours in a Student's *t* test.

**Figure 1** Correlation between percentage BrdU positive cells (labelling index; LI) and S-phase fraction (SPF) for all patients with full analysis ($n = 38$). Correlation coefficient $r = 0.77$.

USPF = (SPF-LI)/SPF. This formula was applied to the population at large (method 1) or to a representative portion of the population by restricting the analysis to a small segment in the second part of the S-phase (method 2). The latter method avoids the possible disturbing effect of cells that are unlabelled because they entered the S-phase in the time period between *in vivo* labelling and bronchoscopy.

Results of the two methods are given in Table III. It appears that the two methods to estimate the USPF are different in that method 2 gives higher estimates. However, in the 3 groups of patients the results of the two methods show a similar tendency; the correlation coefficient was found to be 0.7 ($P = 5.9E\text{-}5$). Whatever method used, the USPF appears to be lower in malignant samples ($P = 0.01$ (method 1) or $P = 0.0064$ (method 2)).

### Discussion

The main goal of this study was to investigate the feasibility of determining cytokinetic characteristics from human bronchoscopy specimens. Therefore we entered a heterogeneous group of patients encompassing the full spectrum of types and stages of bronchial carcinoma as well as biopsies of normal bronchial tissue. Fifty of such specimens were adequate to the extent that patients were eligible and a nuclear suspension of good enough quality for flow cytometric analysis was obtained. Full analysis was possible in 76% of all cases. The main reasons for not being able to perform cytokinetic analysis was the failure to detect a coherent cohort of labelled cells. In these samples very few cells (less than 1%) were found labelled with BrdU and these cells were randomly scattered over the dotplot. This occurred in 6/17 non-malignant and in 6/33 malignant specimens, indicating that successful analysis is possible in an even higher percentage of patients with malignant tumours. Acute side-effects were not observed with the dose of BrdU employed, as has also been the case in earlier studies with this dose and slightly higher doses (Forster *et al.*, 1992; Hoshino *et al.*, 1985; Hupperets *et al.*, 1986; Miller *et al.*, 1991; Nagashima *et al.*, 1988; Shimomatsuya *et al.*, 1991; Ten Velde *et al.*, 1989*a*; Wilson *et al.*, 1988).

During the course of this study, some problems have arisen concerning flow cytometric analysis. Difficulties in discerning the tumour cell population from other cell types present in the bronchoscopy sample were manifest in particular in diploid samples, where it is not possible to make a distinction between normal cells or tumour cells with a diploid DNA content. In aneuploid samples, the major problem was overlap of diploid and aneuploid populations. An additional disturbing effect was generated by the accumulation of cell debris in the sample. This makes estimations of cell numbers in various phases of the cell cycle less reliable. In order to avoid the problems mentioned above, we started to investigate the use of dual parameter image cytometry as an alternative. This technique is currently being optimised. Begg *et al.* (1991) recently described three-colour fluorescence flow cytometry as a method for selecting the desired malignant cell population for kinetic analysis, that also may be useful in this respect.

One of the most striking results of this study is the finding of a considerable number of cells with a DNA content between G1 and G2 that do not take up BrdU: the so-called unlabelled S-phase cells. These cells were observed in virtually each sample. Previously, the existence of this phenomenon had also been described by Darzynkiewicz (1986), Forster *et al.* (1992), Ten Velde *et al.* (1989*a* and *b*) and Wilson *et al.* (1988), while an exceeding of the SPF over the LI was reported by Meyer & Coplin (1988); Teodori *et al.* (1990) and Wilson *et al.* (1985). We were concerned about technical artefacts and therefore determined the frequency of such cells (USPF) by restricting the analysis to a window close to the G2/M peak. This eliminates the contribution of cells that have entered the S-phase in the time between labelling with BrdU and sampling of the biopsy, and would also possibly decrease the influence of debris. Still, we found that 36–99% of the S-phase cells were unlabelled. In inter-

**Figure 2** Aneuploid tumour biopsy sample. X-axis: DNA content; Y-axis: BrdU fluorescence.

preting these data it has to be taken into account, however, that cell debris may still interfere with the estimation of the USPF, in particular in diploid samples with a relatively low frequency of labelled cells. This is due to the fact that cell debris shows an exponential decline with relatively higher counts in the lower DNA channels. This may in part explain why normal tissues with a low LI were found to have a higher USPF as compared to malignant biopsies.

The cause of S-phase cells not to incorporate the DNA precursor BrdU can be 2-fold: either the cells are cycling but the BrdU cannot reach them of the BrdU is present but the cells do not cycle. The first possibility implies dose-related problems. In a study with human bone marrow, Hupperets *et al.* (1986) investigated the feasibility of *in vivo* labelling of bone marrow cells with BrdU in doses varying from 700 mg $m^{-2}$ to 50 mg $m^{-2}$. They found that *in vivo* labelling with 50 mg $m^{-2}$ BrdU was safe, feasible and as effective as higher doses. Furthermore, if dose was an issue, one would expect to find cells with a labelling intensity varying between negative and maximally positive rather than two distinct populations with clearly negative and positive characteristics, as we observed and as is illustrated in Figure 2. Finally, Forster *et al.* (1992), using a higher dose of BrdU in a study of squamous head and neck cancer, reported similar labelling indices as well as the presence of unlabelled S-phase cells.

Morstyn *et al.* (1983) found a uniform BrdU distribution in small melanoma, but a heterogeneous distribution of BrdU in large melanoma which they ascribed to differences in perfusion. This points to the possibility that the presence of unlabelled S-phase cells is related to local dose problems, due to the presence of areas with poor perfusion. In that case, one would expect the cells that do label to pass through the S-phase with a constant rate. In contrast, we found variable durations of S-phase with some biopsies showing extremely long S-phase transit times. It cannot be ruled out, however, that at least part of the S-phase cells do not label because of poor availability of BrdU.

A second possibility to explain the presence of unlabelled S-phase cells could be that cells, for some reason, stop cycling during S-phase. Assuming that this is a random event, this would imply that cells in the BrdU labelled population will arrest also. According to the theoretical model developed by White (1991), this will result in high values for Ts. Therefore, the length of the S-phase transit time could be another indicator for the presence of unlabelled S-phase cells. From the data presented in Table III, it appears that although the mean S-phase transit time did not differ between the various groups, there always was a wide range with some samples showing very long Ts. It remains to be determined whether the duration of S-phase could be a better indicator for the occurrence of cell arrest in S-phase than the fraction of unlabelled S-phase cells, in particular in case of a poor ratio between the degree of labelling and the presence of debris. The background and clinical implications of unlabelled S-phase cells are currently being investigated by performing *in vitro* experiments subjecting tumour cells to poor metabolic states.

When comparing various groups of samples, we found significant differences in SPF, LI and potential doubling time between malignant and non-malignant biopsies. Within the various types of lung cancer, cytokinetic parameters did not differ. Similarly, no significant differences were observed between the two major groups of lung cancer; small cell lung cancer (SCLC) and non small cell lung cancer (NSCLC). In particular, potential doubling times did not differ (SCLC: 8.6 days; NSCLC: 8.1 days), whereas small cell lung tumours are known to be clinically more aggressive and appear to double their cell number at a higher rate than do cancers of the non small cell type. This points to the possibility that the cell loss factor is considerably higher in non small cell lung cancer than in small cell lung cancer. The mean tumour volume doubling times for these types of lung cancer are approximately 3 months (100 days) for NSCLC and 1 month (30 days) for SCLC (Selawry & Hansen, 1982). Our cytokinetic data however, indicate a doubling of the cell number in 8.1 days (NSCLC) and 8.6 days (SCLC). Wilson *et al.* (1988), who calculated the potential tumour doubling time in 25 solid human tumours, found an overall mean of only 5.5 days. A significant discrepancy between the calculated tumour doubling time and the measured doubling time was also reported by Terz *et al.* (1971). The cell loss factor can be defined as $(1-(T_{pot}/T_d)) \times 100\%$ (Steel, 1977), where $T_{pot}$ is the potential doubling time (assuming there is no cell loss) and $T_d$ is the actual doubling time. Using this formula and our data, the cell loss factor in NSCLC is estimated as to be 90%, whereas the cell loss in SCLC is estimated on 70%. This is in accord with one previous report, in which the cell loss of human tumours was found to range from 40% to 80%, with a higher cell loss in squamous cell lung carcinoma than in small cell lung carcinoma (Shimomatsuya *et al.*, 1991). Taken together, our data strongly support the concept that the relatively slow growth rate of NSCLC as compared to SCLC does not depend on differences in proliferation but rather on the rate of cell loss.

Reports in the literature, concerning Ki67 expression, show no significant differences between SCLC and NSCLC, although there seems to be a trend towards a higher positive Ki67 fraction in SCLC (Gatter *et al.*, 1986, Soomroo & Whimster, 1990). This is not in conflict with our hypothesis that SCLC exhibit a lower cell loss factor than NSCLC, since Ki67 is a protein which is expressed in proliferating cells. However, it is a static marker which does not reflect active proliferation and which therefore cannot be directly compared to the cytokinetic parameters measured in this study.

We have demonstrated the feasibility of obtaining dynamic cytokinetic data from bronchoscopy specimens after *in vivo* labelling with BrdU in a majority of patients presenting with lung cancer. Growth parameters such as the potential tumour doubling time, the S-phase transit time and an estimation of the presence of arrested S-phase cells can be obtained that will now be assessed for their prognostic and therapeutic implications.

This study was supported by a grant from the Dutch Cancer Society (grant no. IKL-90-01).

### References

BAISCH, H., BECK, H., CHRISTENSEN, I., HARTMANN, N., FRIED, J., DEAN, P., GRAY, J., JETT, J., JOHNSTON, D., WHITE, R., NICOLINI, C., ZIETZ, S. & WATSON J. (1982). A comparison of mathematical methods for the analysis of DNA histograms obtained by flow cytometry. *Cell Tissue Kinet.*, **15**, 235–249.

BEGG, A.C. & HOFLAND, I. (1991). Cell kinetic analysis of mixed populations using three-color fluorescence flow cytometry. *Cytometry*, **12**, 445–454.

BEGG, A.C., MCNALLY, N.J., SCHRIEVE, D.C. & KARCHNER, H. (1985). A method to measure the duration of DNA synthesis and the potential doubling time from a single sample. *Cytometry*, **6**, 620–626.

CARLTON, J.C., TERRY, N.H.A. & WHITE, R.A. (1991). Measuring potential doubling times of murine tumors using flow cytometry. *Cytometry*, **12**, 645–650.

DARZYNKIEWICZ, Z. (1986). Cytochemical probes of cycling and quiescent cells applicable to flow cytometry. In *Techniques in Cell Cycle Analysis*, Gray, J.E. & Darzynkiewicz, Z. (eds) pp. 255–290. Human Press, Inc: Clifton, New Jersey.

FORSTER, G., COOKE, T.G., COOKE, L.D., STANTON, P.D., BOWIE, G. & STELL, P.M. (1992). Tumor growth rates in squamous carcinoma of the head and neck measured by *in vivo* bromodeoxyuridine incorporation and flow cytometry. *Br. J. Cancer*, **65**, 698–702.

FRAIRE, A.E., JOHNSON, E.H., YESNER, R., ZHANG, X.B., SPJUT, H.J. & GREENBERG, S.D. (1992). Prognostic significance of histopathologic subtype and stage in small cell lung cancer. *Human pathol.*, **23**, 520–528.

GATTER, K.C., DUNNILL, M.S., GERDES, J., STEIN, H. & MASON, D.Y. (1986). New approach to assessing lung tumours in man. *J. Clin. Pathol.*, **39**, 590–593.

HIDDEMAN, W., SCHUMANN, J., ANDREEFF, M., BARLOGIE, B., HERMAN, C.J., LEIF, R.C., MAYALL, B.H., MURPHY, R.F. & SANDBERG, A.A. (1984). Convention on nomenclature for DNA cytometry. *Cytometry*, **5**, 445–446.

HOSHINO, T., NAGASHIMA, T., MUROVIC, J., LEVIN, E.M., LEVIN, V.A. & RUPP, S.M. (1985). Cell kinetic studies of *in situ* human brain tumors with bromodeoxyuridine. *Cytometry*, **6**, 627–632.

HUPPERETS, P.S.J.G., SCHUTTE, B., VAN ASSCHE, C., REIJNDERS, M.M.J. & BLIJHAM, G.H. (1986). Proliferative characteristics of human bone marrow cells following *in vivo* administration of bromodeoxyuridine. *Cancer Chemother. Pharmacol.*, **18**, suppl. 1, 148.

KALLIONIEMI, O.P., HIETANEN, T., MATTILA, J., LEHTINEN, M., LAUSLAHTI, K. & KOIVULA, T. (1986). Aneuploid DNA content and high S phase fraction of tumour cells are related to poor prognosis in patients with primary breast cancer. *Eur. J. Cancer Clin. Oncol.*, **23**, 277.

MEYER, J.S. & COPLIN, M.D. (1988). Thymidine labelling index, flow cytometric S-phase measurement, and DNA index in human tumors. *Am. J. Clin. Path.*, **89**, 586–595.

MILLER, M.A., MAZEWSKI, C.M., YOUSUF, N., SHEIKH, Y., WHITE, L.M., YANIK, G.A., HYAMS, D.M., LAMPKIN, B.C. & RAZA, A. (1991). Simultaneous immunohistochemical detection of IUdR and BrdU infused intravenously to cancer patients. *J. Histochem. Cytochem.*, **39**, 407–412.

MORSTYN, G., HSU, S.-M., KINSELLA, T., GRATZNER, H., RUSSO, A. & MITCHELL, J.B. (1983). Bromodeoxyuridine in tumors and chromosomes detected with a monoclonal antibody. *J. Clin. Invest.*, **72**, 1844–1850.

NAGASHIMA, T., HOSHINO, T., CHO, K.G., EDWARDS, M.S.B., HUDGINS, R.J. & DAVIS, R.L. (1988). The proliferative potential of human ependymomas measured by *in situ* bromodeoxyuridine labeling. *Cancer*, **61**, 2433–2438.

O'REILLY, S.M., CAMPLEJOHN, R.S., BARNES, D.M., MILLIS, R.R., ALLEN, D., RUBENS, R.D. & RICHARDS, M.A. (1990). DNA index, S-phase fraction, histological grade and prognosis in breast cancer. *Br. J. Cancer*, **61**, 671–674.

SCHUTTE, B., REIJNDERS, M.M.J., VAN ASSCHE, C.L.M.V.J., HUPPERETS, P.S.J.G., BOSMAN, F.T. & BLIJHAM, G.H. (1987). An improved method for the immunocytochemical detection of bromodeoxyuridine labeled nuclei using flow cytometry. *Cytometry*, **8**, 372–376.

SELAWRY, O.S. & HANSEN, H.H. (1982). Respiratory tract cancer. In *Cancer Medicine*, Holland, J.F. & Frei, E. (eds) pp. 1732, Lea & Febiger: Philadephia.

SHIMOMATSUYA, T., TANIGAWA, N. & MURAOKA, R. (1991). Proliferative activity of human tumors: assessment using bromodeoxyuridine and flow cytometry. *Jpn. J. Cancer Res.*, **82**, 357–362.

SILVESTRINI, R., MUSCOLINO, G., COSTA, A., LEQUAGLIE, C., VENERONI, S., MEZZANOTTE, G. & RAVASI, G. (1991). Could cell kinetics be a predictor of prognosis in non-small cell lung cancer? *Lung cancer*, **7**, 165–170.

SOOMROO, I.N. & WHIMSTER, W.F. (1990). Growth fraction in lung tumours determined by Ki67 immunostaining and comparison with AgNOR scores. *J. Pathol.*, **162**, 217–222.

STEEL, G.G. (1977). *Growth kinetics of tumours*, pp. 70–72. Clarendon Press: Oxford.

TERZ, J.J., CURUTCHET, H.P. & LAWRENCE, W. (1971). Analysis of the cell kinetics of human solid tumors. *Cancer*, **5**, 1100–1110.

TEODORI, L., TRINCA, M.L., GOEHDE, W., HEMMER, J., SALVATI, F., STORNIELLO, G. & MAURO, F. (1990). Cytokinetic investigation of lung tumors using the anti-bromodeoxyuridine (BUdR) monoclonal antibody method: comparison with DNA flow cytometric data. *Int. J. Cancer*, **45**, 995–1001.

TEN VELDE, G.P.M., SCHUTTE, B., VERMEULEN, A., VOLOVICS, A., REIJNDERS, M.M.J. & BLIJHAM, G.H. (1988). Flowcytometric analysis of DNA ploidy level in paraffin embedded tissue of non-small lung cancer. *Eur. J. Cancer Clin. Oncol.*, **24**, 455–460.

TEN VELDE, G.P.M. (1989*a*). Cytokinetic analysis of lung cancer specimens by *in vivo* bromodeoxyuridine labeling. In *Thesis.* pp. 85–94. University of Limburg: Maastricht, The Netherlands.

TEN VELDE, G.P.M., SCHUTTE, B., REIJNDERS, M.M.J., BOSMAN, F.T. & BLIJHAM, G.H. (1989*b*). Cytokinetic analysis of lung cancer by bromodeoxyuridine labeling of cytology specimens. *Cytometry*, **10**, 807–810.

TUBIANA, M. & COURDI, A. (1989). Cell proliferation kinetics in human solid tumors: relation to probability of metastatic dissemination and long-term survival. *Radiother. Oncol.*, **15**, 1–18.

VOLM, M., MATTERN, J., SONKA, J., VOGT-SCHADEN, M. & WAYSS, K. (1985). DNA distribution in non-small-cell lung carcinomas and its relationship to clinical behavior. *Cytometry*, **6**, 348–356.

WHITE, R.A. (1991). A theory for analysis of cell populations with non-cycling S phase cells. *J. Theor. Biol.*, **150**, 201–214.

WHITE, R.A., TERRY, N.H.A., MEISTRICH, M.L. & CALKINS, D.P. (1990). Improved method for computing potential doubling time from flow cytometric data. *Cytometry*, **11**, 314–317.

WILSON, G.D., MCNALLY, N.J., DUNPHY, E., KARCHER, H. & PFRAGNER, R. (1985). The labelling index of human and mouse tumours assessed by bromodeoxyuridine staining in vitro and in vivo and flow cytometry. *Cytometry*, **6**, 641–647.

WILSON, G.D., MCNALLY, N.J., DISCHE, S., SAUNDERS, M.I., DES ROCHERS, C., LEWIS, A.A. & BENNETT, M.H. (1988). Measurement of cell kinetics in human tumours in vivo using bromodeoxyuridine incorporation and flow cytometry. *Br. J. Cancer*, **58**, 423–431.